# Fe-Based Amorphous Magnetic Powder Cores with Low Core Loss Fabricated by Novel Gas–Water Combined Atomization Powders

**DOI:** 10.3390/ma15186296

**Published:** 2022-09-10

**Authors:** Jiaqi Liu, Yannan Dong, Zhengqu Zhu, Huan Zhao, Jing Pang, Pu Wang, Jiaquan Zhang

**Affiliations:** 1 School of Metallurgical and Ecological Engineering, University of Science and Technology Beijing, Beijing 100083, China; 2 Qingdao Yunlu Advanced Materials Technology Co., Ltd., Qingdao 266232, China

**Keywords:** gas–water combined atomization, amorphous powders, magnetic powder cores, core loss, high-frequency properties

## Abstract

FeSiBCCr amorphous powders were produced by a novel gas–water combined atomization process, and the corresponding MPCs (magnetic powder cores) were subsequently fabricated by phosphating treatment (0.4~1.6 wt.%), cold pressing (550~2350 MPa), and annealing (423~773 K), respectively. The results showed that the powders had high circularity, excellent thermal stability (ΔT = 59 K), and high saturation magnetization (0.83 T), which could provide raw powders for high-performance MPCs. With increasing phosphoric acid concentrations, despite the increase in DC-bias%, the uniformity of the insulation layers deteriorated, which led to a decrease in permeability and an increase in core loss. With increasing compaction pressures, the core loss increased continuously, and the permeability and DC-bias% first increased and then decreased. When annealing below the crystallization temperature, with increasing annealing temperatures, the permeability increased, and the core loss and DC-bias% decreased continuously. Under the optimized process of 0.4 wt.% phosphating concentration, 550 MPa pressure, and 773 K annealing temperature, the MPCs had a permeability of 21.54 ± 1.21, DC-bias% of 90.3 ± 0.2, and a core loss (*B*_m_ = 50 mT, *f* = 100 kHz) of 103.0 ± 26.3 mW cm^−3^. The MPCs had excellent high-frequency low-loss characteristics and showed great application potential under the development trends of high current, high power, and high frequency of electronic components.

## 1. Introduction

As important basic electromagnetic components, magnetic powder cores (MPCs) are widely used in switched-mode power supplies, inductors, electro mobiles, medical equipment, and electro-communication [1,2,3]. Under the strategic goal of “energy conservation and emission reduction”, miniaturization, high efficiency, and high frequency are the inevitable trends in the development of electronic devices, which puts forward higher requirements for the soft magnetic properties of MPCs under high-frequency conditions [4,5]. Currently, Fe-based amorphous powders have become ideal materials for MPCs applying at high frequency due to unique advantages such as high *M*_s_ (saturation magnetization), low *H*_c_ (coercivity), low *P*_cv_ (core loss), and low cost [5,6,7]. The properties of MPCs, such as permeability, *P*_cv_, and DC bias performance, are often closely related to the raw powders [8]. The fine powders can improve the compact density and the high-frequency performance of the MPCs. The circularity of powders directly affects the uniformity of the insulation layers. Irregularly shaped particles with sharp edges are not conducive to the formation of uniform and dense insulation layers, resulting in deterioration of the magnetic properties. The excessive volume fraction of crystalline phases in raw powders will lead to a sharp increase in *H*_c_, and thus *P*_cv_ will be greatly increased [9,10]. Therefore, fine spherical amorphous powders with excellent soft magnetic properties are of great significance for the fabrication of high-performance MPCs and new-generation electronic components.

The main processes used to produce amorphous powders include ribbon pulverization, mechanical alloying, and atomization [9,11]. The ribbon pulverization and mechanical alloying both prepare amorphous powders by mechanical crushing of raw materials. Therefore, the circularity of powders is extremely poor, which makes it difficult to form uniform insulation layers on the powder surfaces and leads to a significant increase in *P*_cv_. In addition, the smaller demagnetization factor of the powders leads to a decrease in the resonance frequency, resulting in the deterioration of high-frequency performance [8]. The atomization method can be divided into gas atomization (GA), water atomization (WA), and gas–water combined atomization (CA) according to the atomization medium. During the GA process, the molten metal is broken by inert gas, and the cooling rate is usually between 10^2^ and 10^3^ K s^−1^, which requires high GFA (glass forming ability) for the amorphous alloy system, but the droplets have sufficient time to complete the spheroidization process due to a relatively slow solidification rate, and thus the circularity of GA powders is high [10,12]. In the process of WA, the molten melt is directly impacted by high-pressure water jets, which have a stronger cooling capacity due to the larger specific heat of water, and the cooling rate is usually in the range of 10^3^~10^4^ K s^−1^, which makes it easy to fabricate amorphous powders even on an industrial scale, but the intense jet impact and too fast cooling rate lead to rough surfaces and inadequate spheroidization of WA powders [1,12,13]. During the CA process, spherical droplets are first obtained by GA, and then the droplets are rapidly cooled by WA to fabricate amorphous powders with high circularity [9]. In summary, the CA process has unique advantages such as a rapid cooling rate and excellent properties of prepared powders, and it has become one of the frontier processes for the industrial production of amorphous powders.

MPCs are fabricated from powders after insulated coating, consolidation, and annealing [3]. Insulated coating is one of the key steps to improving the high-frequency properties of MPCs. The powder resistivity can be increased from the order of mΩ·cm to the order of 10^6^~10^8^ Ω·cm by uniform and dense insulation layers, thereby effectively reducing the eddy current loss under high frequency [14,15]. Phosphating treatment is a widely used insulated coating method, which can effectively remove impurities on the powder surfaces and form phosphate films, improve the resistivity of MPCs, and reduce *P*_cv_. In addition, for the subsequent organic insulated coating, the phosphate layer is also an excellent transition layer, which can effectively improve the uniformity of insulation layers [16,17,18]. The coated powders can be consolidated by cold pressing, warm pressing, spark plasma sintering, and so on, followed by annealing to release the internal stress accumulated in the MPCs during the powder consolidation process and improve the comprehensive magnetic properties [5].

At present, research on improving the performance of MPCs has been widely reported. Chang et al. prepared MPCs using FeSiBP GA powders and the comprehensive magnetic properties of MPCs were effectively improved by optimizing the insulated coating and heat treatment parameters [18]. Liu et al. fabricated FePBSiNbCr MPCs by WA amorphous powders and the effect of epoxy resin (EP) contents on the performance of MPCs were investigated [19]. Zhao et al. compared the comprehensive properties of powders and MPCs prepared by WA, GA, and CA. They found that powders prepared by the CA process had excellent overall properties and the corresponding MPCs also had high permeability, low *P*_cv_, and excellent DC bias performance [9]. Xia et al. mixed FeSiCr amorphous powders with carbonyl iron powders and prepared corresponding soft magnetic composites, and the results showed that the proper addition of carbonyl iron powders could effectively improve the permeability and reduce *P*_cv_ [20]. Yan et al. used mechanical ball milling to coat SiO_2_ nanoparticles on the surfaces of FeSiB amorphous powders and prepared MPCs with excellent soft magnetic properties under high frequency by spark plasma sintering [6]. Long et al. prepared polyimide-phosphate double coating FeSiCr MPCs and *P*_cv_ under high frequency was effectively reduced by optimizing the annealing temperature [17].

In this study, Fe_70.8_Si_11.4_B_11.9_C_3.2_Cr_2.7_ (at.%) amorphous powders were prepared by a novel CA process, and the corresponding MPCs were fabricated by cold pressing and annealing after phosphating treatment. The effects of phosphoric acid concentrations, compaction pressures, and annealing temperatures on the magnetic properties of MPCs were investigated, which could provide theoretical and practical guidance for the development of new generation high-performance MPCs for high current and high-frequency applications.

## 2. Materials and Methods

### 2.1. Preparation of FeSiBCCr Amorphous Powders

**Figure 1:**
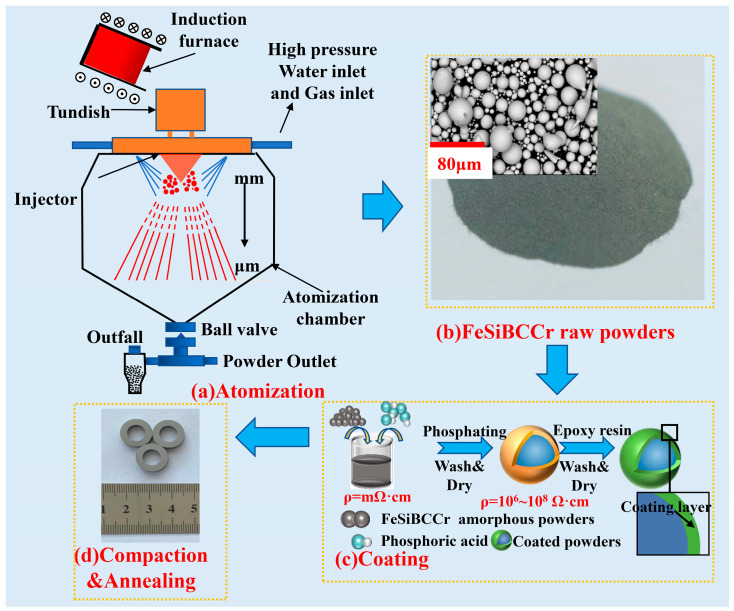
Schematic diagram of fabrication process for FeSiBCCr MPCs.

The schematic diagram of the novel CA process used in this work is shown in Figure 1a. According to the composition of Fe_70.8_Si_11.4_B_11.9_C_3.2_Cr_2.7_ (at.%), iron (purity > 99.9%), silicon (purity > 99.5%), boron (purity > 99.9%), chromium (purity > 99.0%), and pre-alloyed Fe-B and Fe-C alloys were mixed and heated to 1773 K in a vacuum induction melting furnace with a capacity of 15 kg. Then, the melt in the furnace was poured into a tundish equipped with thermal insulating equipment, and the temperature of the melt in the tundish was controlled at 1273 K. After the preparation stage of N_2_ atmosphere in the atomization chamber was completed, the melt was injected into the atomization chamber through a delivery tube with a diameter of 2.0 mm and was sequentially cooled by N_2_ jets and high-pressure water jets. The atomization gas pressure was 1.7 MPa, the water flow rate was 90 L·min^−1^, and the water velocity was 80 m·s^−1^. The powders were dried and sieved by a standard sieve with 300 meshes (<48 μm) to obtain the raw powders as shown in Figure 1b.

### 2.2. Preparation of MPCs

As shown in Figure 1c, the raw powders were passivated by phosphoric acid solutions, and placed in a stirrer with 350 rpm for 30 min. After phosphate treatment, the powders were cleaned by acetone solutions 3 times, and then the powders were added into 0.5 wt.% rust inhibitor solutions, and stirred with a glass rod for 10 min. After that, the powders were mixed with 3 wt.% EP in a stirrer with 300 rpm for 30 min, and dried at 343 K for 30 min. The coated powders were put into a mold of φ_1_ 14 mm × φ_2_ 8 mm × h 3.5 mm (outer diameter 14 mm, inner diameter 8 mm, height 3.5 mm) for cold pressing for 30 s, and finally annealed for 2 h to improve the comprehensive properties of MPCs (as shown in Figure 1d).

In order to study the effects of phosphoric acid concentrations, compaction pressures (*P*_c_), and annealing temperatures (*T*_a_) on the soft magnetic properties of MPCs, three groups of experimental schemes as shown in Table 1 were adopted, respectively. Among them, except Group B, 3 MPCs were fabricated under each condition in Group A and Group C.

### 2.3. Material Characterization

The size distribution of the raw powders was measured using the laser-diffraction particle-size analyzer (BT-9300S, Bettersize Instruments Ltd., Dandong, China). The detection particle size range was 0.1~341 μm, the shading rate was set as 16.02%, the refractive index of the medium was set as 1.333, and the refractive index of the sample was set as 2.860. The internal structure of the raw powders and the annealed powders was analyzed by the X-ray diffraction (XRD, D2 PHASER, BRUKER AXS, Karlsruhe, Germany) using Cu *K*_α_ radiation with λ = 0.154184 nm. The step size was 0.02°, the scanning range was 20~100°, the tube voltage was 30 kV, and the tube current was 10 mA. The morphology of the raw powders and coated powders was observed by a scanning electron microscope (SEM, Phenom Pro Desktop SEM, Phenom-World BV, Eindhoven, The Netherlands). The circularity of the raw powders was calculated by Phenom Prisuite Software v2.9.0 (Phenom-World BV, Eindhoven, The Netherlands). The characteristic temperatures of the raw powders were measured by the differential scanning calorimeter (DSC, Setaram Setsys Evo, KEP Technologies, Lyon, France) at a heating rate of 10 K·min^−1^ and a 30 mL·min^−1^ flow rate of high purity argon. The hysteresis loop of the raw powders were measured by the vibrating-sample magnetometer (VSM, Lake Shore 8604, Lake Shore Cryotronics, Inc., Westerville, OH, USA) at the maximum applied magnetic field intensity of ± 8.0 × 10^5^ A·m^−1^ [5]. Additionally, the frequency was 50 Hz. The density of MPCs was obtained by dividing actual mass by volume. The total core losses under the condition of *B*_m_ = 50 mT, *f* = 100 kHz and *B*_m_ = 20 mT, *f* = 1 MHz were measured by a B-H curve analyzer (SY-8219, IWATSU ELECTRIC Co., Ltd., Tokyo, Japan). The effective permeability (*μ_e_*) and DC bias were obtained using a LCR meter (3265B, WAYNE KERR Electronics, Bognor Regis, UK). The effective permeability was calculated by the following equation [9]:

(1) $$\mu_{e}=\frac{Ll_{e}}{\mu_{0}N^{2}A_{e}}$$

where *μ_e_* is the effective permeability; *L* is the inductance of MPCs; *l*_e_ is the effective magnetic circuit length of MPCs; *N* is the number of copper wires turns (14 in this study); *A*_e_ is the effective cross-section area of MPCs; *μ*_0_ is the permeability of a vacuum.

## 3. Results

### 3.1. Characterization of Atomization Powder

**Figure 2:**
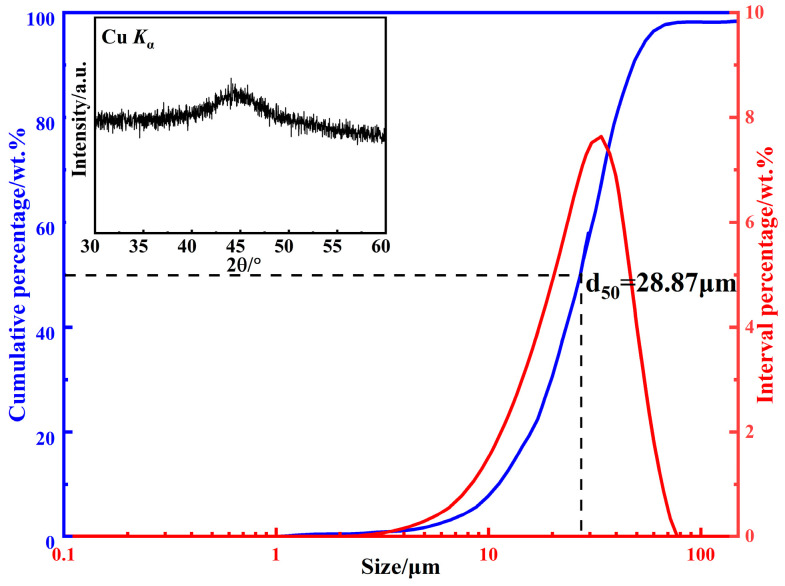
Size distribution and XRD pattern of FeSiBCCr amorphous powders.

The size distribution of the raw powders is shown in Figure 2. The powder size shows a unimodal distribution, *d*_50_ is 28.87 μm, and the standard deviation *d*_84_/*d*_50_ is 1.55, indicating that the powder size distribution range is narrow and the large particles exceeding 100 μm are very few. Compared with large particles, small particles have difficulty in forming an easy magnetization axis due to the regular shape, and then the permeability is lower, but the high-frequency stability can be significantly improved. In addition, the fine powders can not only improve the density of the prepared MPCs, but also reduce the skin depth, thereby reducing the eddy current loss at high frequency [21,22]. The XRD pattern of CA powders is shown in the inset of Figure 2. No sharp diffraction peaks corresponding to the crystalline phases can be observed, and a typical broad peak of the amorphous phase appears near the 45° diffraction angle, indicating that the CA powders are amorphous, and also proving that the novel CA process in this study has an extremely fast cooling rate.

**Figure 3:**
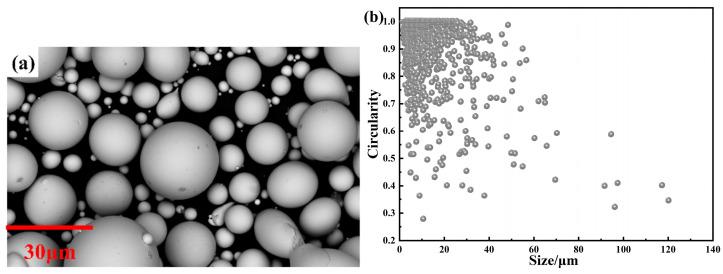
(**a**) SEM pictures of FeSiBCCr amorphous powders; (**b**) variation in circularity with size of powders.

It can be seen from the SEM pictures in Figure 3a that the powder surfaces are smooth, most of the powders are nearly spherical, and have no obvious crystal structures. Combined with the XRD results, it can be considered that the raw powders are completely amorphous. Figure 3b shows the relationship between the size and circularity of the powders obtained by Phenom Prisuite Software v2.9.0. The small droplets formed by multi-stage break-up during the atomization process have a smaller size and require a shorter time for spheroidization, and so the solidified powders have higher circularity. In contrast, large droplets are mainly formed due to agglomeration between incompletely solidified droplets and rapid solidification of droplets that have not undergone sufficient break-up, and so large particles tend to be less spherical and have irregular shapes [10,23]. In this study, the average circularity of the raw powders is 0.913, indicating that the powders have high circularity. Compared with the irregularly shaped powders, although the permeability of spherical powders is lower due to the larger demagnetization field, the spherical powders have higher resonance frequency and can be applied under higher frequency conditions [10]. Moreover, more uniform and dense insulation layers can be formed during the subsequent insulated coating process to further reduce *P*_cv_ under high frequency [21,22,24,25,26].

**Figure 4:**
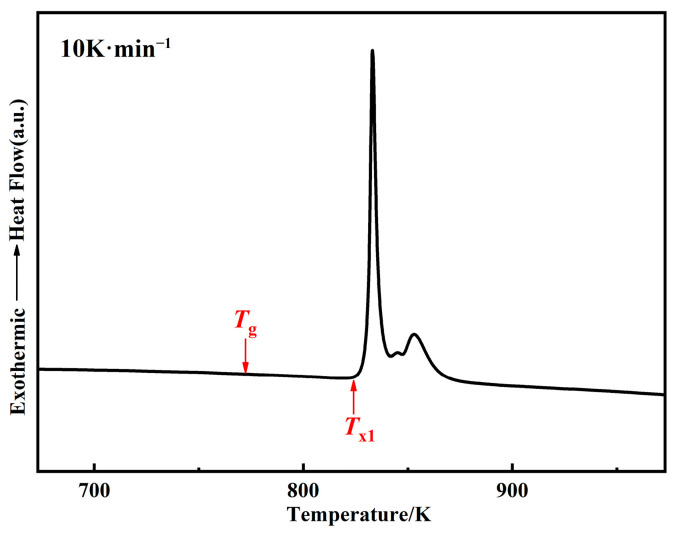
DSC curve of FeSiBCCr amorphous powders (10 K·min^−1^).

Figure 4 shows the DSC curve of the raw powders at the heating rate of 10 K·min^−1^. The results show that three crystallization peaks are formed, which is consistent with a previous study [5]. The glass transition temperature *T*_g_ is 771 K, the onset temperature of the first crystallization peak *T*_x1_ is 830 K, and the undercooled liquid region Δ*T* = *T*_x1_ − *T*_g_ = 59 K could be obtained. Therefore, the FeSiBCCr amorphous system has excellent thermal stability and processing performance and can maintain an amorphous structure with high resistivity and low core loss at higher annealing temperatures [5,27,28].

**Figure 5:**
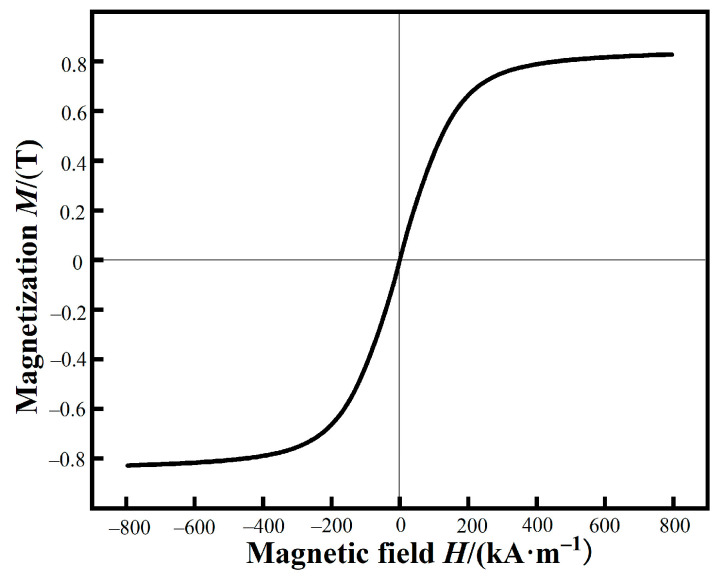
Hysteresis loop of FeSiBCCr amorphous powders.

The hysteresis loop of FeSiBCCr amorphous powders measured by VSM are shown in Figure 5. The *M*_s_ of raw powders are 0.83 T. In our previous work, based on spark plasma sintering, FeSiBCCr bulk amorphous alloys with excellent soft magnetic properties were fabricated using the CA powders [5], which exhibited soft magnetic properties of the CA powders and facilitated the fabrication of high-performance MPCs.

### 3.2. Effects of Phosphoric Acid Concentration on MPC Properties

**Figure 6:**
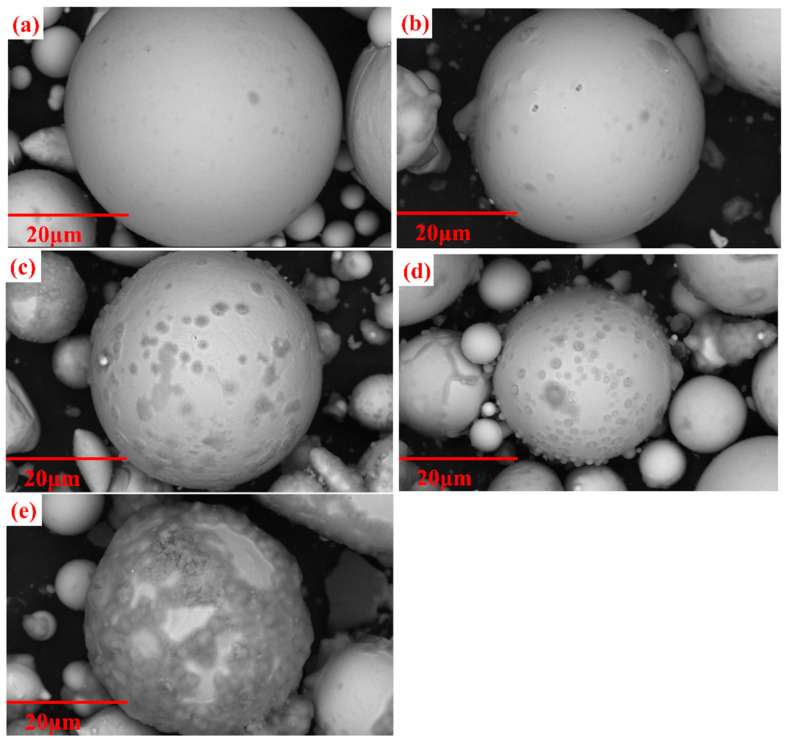
SEM pictures of FeSiBCCr powders treated by different phosphating concentrations: (**a**) 0.4 wt.%; (**b**) 0.6 wt.%; (**c**) 0.8 wt.%; (**d**) 1.2 wt.%; (**e**) 1.6 wt.%.

The SEM pictures of the coated powders by different concentrations of phosphoric acid are shown in Figure 6a–e. As shown in Figure 6a, when the phosphating concentration is 0.4 wt.%, the surface of the powder is smooth, and a uniform phosphate film can be observed. In Figure 6b–d, as the phosphating concentration is increased from 0.6 to 1.2 wt.%, the phosphate nanoparticles that accumulated on the powder surface gradually increase, and the size of the nanoparticles also gradually increases. In Figure 6e, when the phosphating concentration is 1.6 wt.%, the powders are severely corroded, and a thick phosphate layer is formed on the surface, accompanied by particle agglomeration. The effect of different phosphoric acid concentrations on insulated coating is further quantitatively evaluated by characterizing the performance of MPCs in the following.

**Figure 7:**
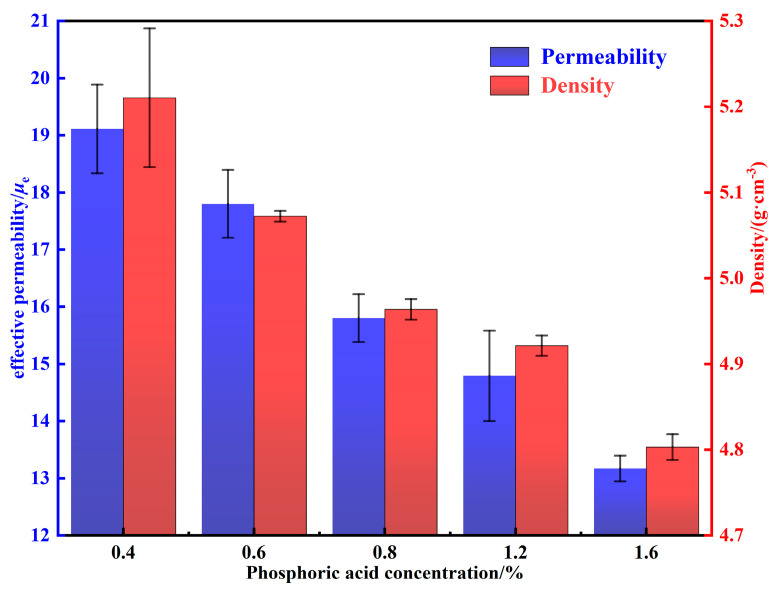
Density and permeability of MPCs fabricated under different phosphoric acid concentrations.

The effective permeability *μ*_e_ of MPCs can be analyzed by the following equation [19]:

(2) $$\mu_{e}=\frac{3+(\mu^{\prime }-1)(3-3g)}{3+g(\mu^{\prime }-1)}$$

where *μ*′ is the permeability of powders; *g* is the content of the non-ferromagnetic phase. *μ_e_* is inversely proportional to the saturation magnetostriction, internal stress, magneto-crystalline anisotropy constant, and nonferromagnetic impurity content of the material [5]. The density and permeability of MPCs treated by different phosphating concentrations are shown in Figure 7. As the phosphoric acid concentration is increased from 0.4 to 1.2 wt.%, the increase in the content of nonferromagnetic phosphate results in a decrease in the permeability of MPCs. In addition, a large number of micro-scale defects such as cracks and pores are formed in the uneven phosphate layers, which further leads to the reduction of density and permeability [16]. When the phosphating concentration is increased from 1.2 to 1.6 wt.%, the phosphate layers are significantly thickened and the agglomerated particles are increased, resulting in a sudden decrease in density and permeability to 4.80 ± 0.01 g·cm^−3^ and 13.17 ± 0.22, respectively.

**Figure 8:**
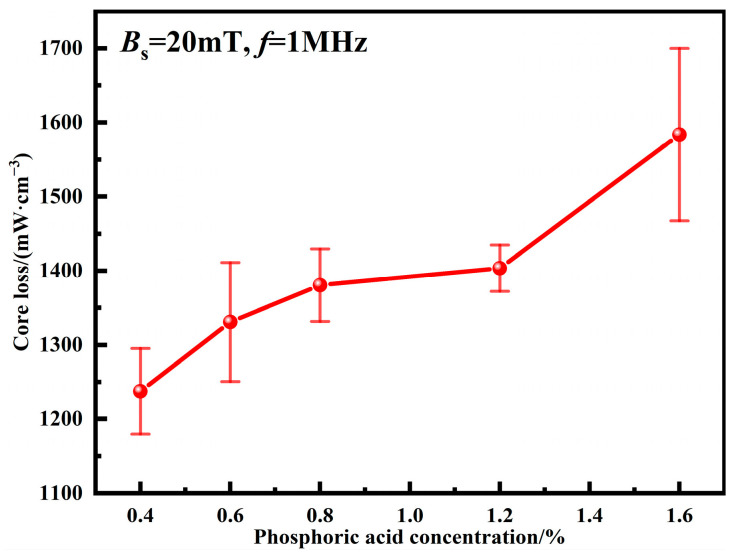
Total core losses of MPCs under different phosphoric acid concentrations.

The total core loss of MPCs can be expressed as the following equation:

(3) $$P_{\mathrm{cv}}=P_{h}+P_{e}=K_{h}f+K_{e}f^{2}$$

where *P*_h_ is the hysteresis loss; *P*_e_ is the eddy current loss; *K*_h_ is the hysteresis loss coefficient; *K*_e_ is the eddy current loss coefficient; *f* is the frequency. *P*_cv_ mainly includes *P*_h_ and *P*_e_. When the frequency is low, *P*_h_ is dominant; when the frequency is high, *P*_e_ begins to dominate. *K*_h_ is generally considered to be mainly proportional to the structure-sensitive physical quantity *H*_c_, which is mainly related to factors such as purity, internal structure, powder size, internal stress, and defects [5,29,30]. *K*_e_ is mainly related to resistivity, which is directly related to the insulated coating. Figure 8 shows the total core losses of MPCs under different phosphating concentrations. It can be seen that *P*_cv_ increases with the increase of phosphoric acid concentrations. When the phosphating concentration is 0.4 wt.%, uniform and dense phosphate layers are formed with the best interparticle insulation, resulting in the lowest *P*_cv_ of 1237.4 ± 58.0 mW cm^−3^ [19]. When the phosphating concentration is increased from 0.4 to 1.2 wt.%, the uniformity of the phosphate layers decreases, air gaps are introduced, and thus *P*_cv_ is increased to 1403.43 ± 31.3 mW cm^−3^ due to the restricted movement of the magnetic domain walls [16]. When the phosphating concentration reaches 1.6 wt.%, the amorphous powders are severely corroded, and the skin depth increases due to the agglomeration of a large number of powders, and so *P*_cv_ is suddenly increased to 1583.6 ± 116.3 mW cm^−3^.

**Figure 9:**
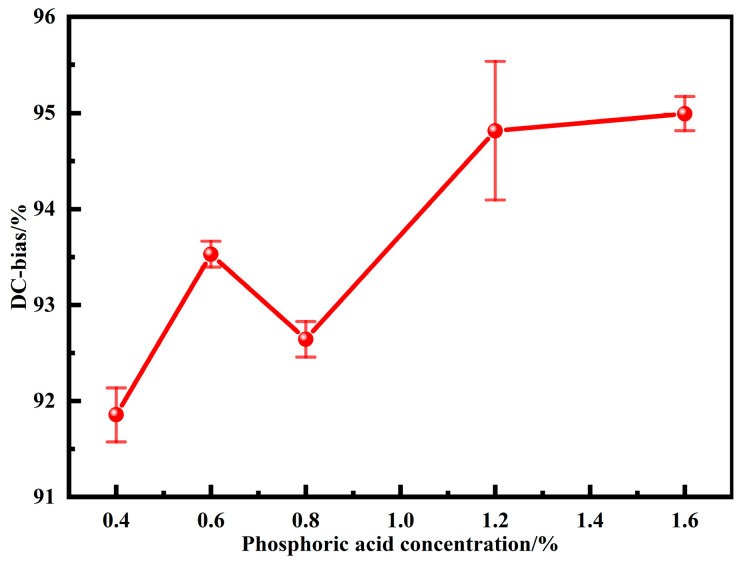
DC bias of MPCs under different phosphoric acid concentrations (DC magnetic field intensity is 71.25 Oe).

Under the action of an external DC magnetic field, the permeability of MPCs inevitably decreases with the increase of the magnetic field intensity. Therefore, under the development trends of high current and miniaturization, the DC bias performance, which directly affects the performance of electronic components under an external magnetic field, is one of the key properties of soft magnetic materials. Figure 9 shows the DC bias performance of MPCs under different phosphoric acid concentrations. The DC-bias% shows an increasing trend as the phosphating concentration is increased from 0.4 to 1.6 wt.%. Combined with the above analysis, it is clear that the thickness of non-ferromagnetic phosphate layers and the content of air gaps increase with phosphating concentrations, which effectively delays saturation magnetization and thus increases the DC-bias% despite the consequent decrease in density and permeability [16,30].

### 3.3. Effects of Compaction Pressures on MPC Properties

**Figure 10:**
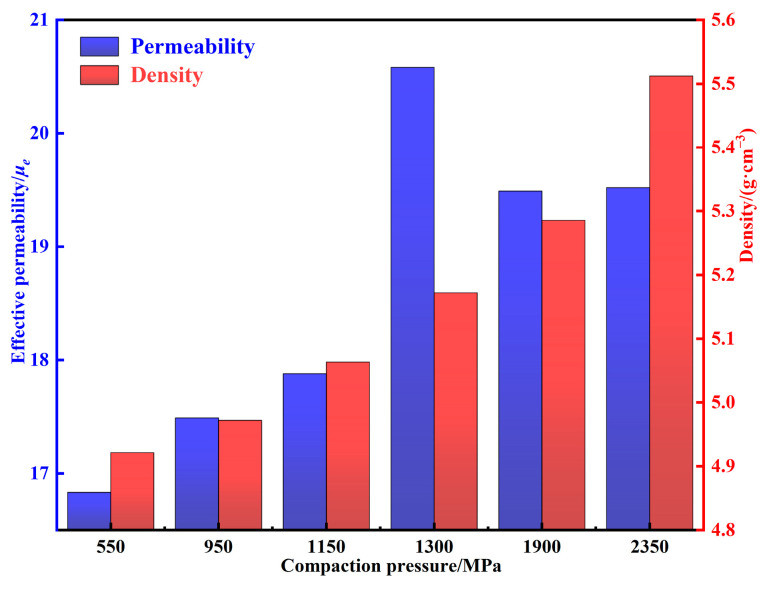
Density and permeability of MPCs under different compaction pressures.

The density and permeability of MPCs under different compaction pressures are shown in Figure 10. With the increase of compaction pressures, the density increases gradually, while the permeability first increases and then decreases. Combined with the discussion in Section 3.2, when the pressure is increased from 550 to 1150 MPa, the density increases from 4.92 to 5.06 g cm^−3^, the content of internal air gaps decreases substantially, the motion resistance to the magnetic domain walls decreases, and thus the permeability increases from 16.83 to 20.58 [31]. However, when the pressure is increased to more than 1150 MPa, pressure that is too large not only accumulates excessive internal stress in the MPCs, which hinders the displacement and rotation of the magnetic domain walls and limits the magnetization process, but also causes the particles to deform along the direction of uniaxial pressure, which leads to the formation of easy magnetization axes and aggravates the magnetic crystal anisotropy [9,32]. Therefore, as the pressure is increased from 1150 to 2350 MPa, the permeability decreases from 20.58 to 19.52.

**Figure 11:**
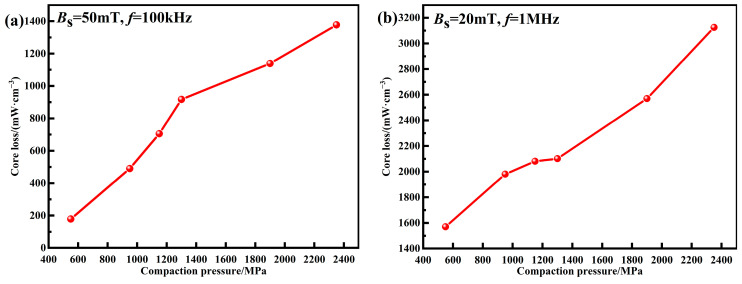
Core losses of MPCs under different compaction pressures: (**a**) *B*_s_ = 50 mT, *f* = 100 kHz; (**b**) *B*_s_ = 20 mT, *f* = 1 MHz.

The total core losses of MPCs prepared under different compaction pressures are shown in Figure 11. For the core loss at *B*_m_ = 50 mT and *f* = 100 kHz, as the pressure is increased, the accumulation of internal stress causes the increase of the hysteresis loss, leading to an increase in *P*_cv_ from 178.6 mW cm^−3^ at 550 MPa to 1377.3 mW cm^−3^ at 2350 MPa. For the core loss at *B*_m_ = 20 mT and *f* = 1 MHz, it also increases with increasing pressure. When the pressure is increased to above 1300 MPa, the breakage of the insulation layers is caused by excessive uniaxial pressure, resulting in decreased particle resistance. Therefore, in addition to the higher hysteresis loss caused by the accumulation of internal stress, the eddy current loss also increases significantly, resulting in a rapid increase in *P*_cv_ from 2101.5 mW cm^−3^ at 1300 MPa to 3125.9 mW cm^−3^ at 2350 MPa.

**Figure 12:**
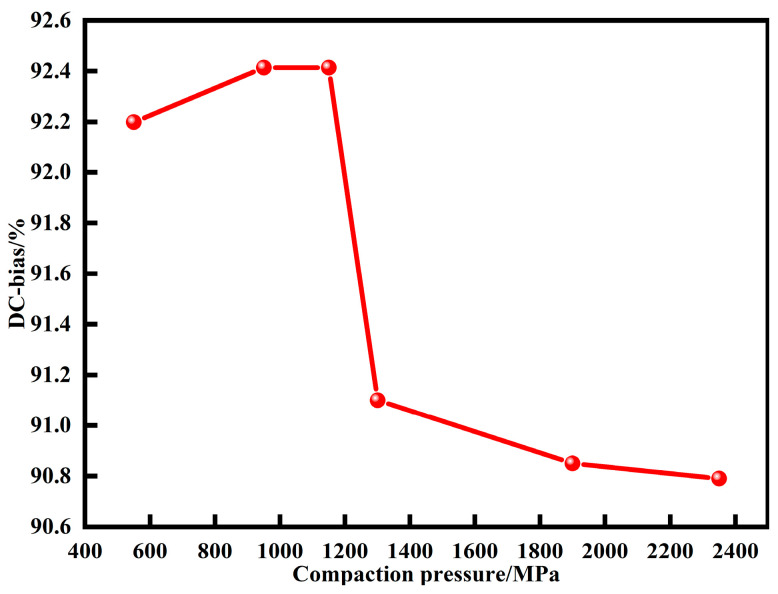
DC bias of MPCs under different compaction pressures (DC magnetic field is 71.25 Oe).

Figure 12 shows the DC bias performance of MPCs under different pressures. With the increase of pressures, DC-bias% first increases and then decreases. Since internal stress accumulation, non-ferromagnetic phase content, and powder shape are important factors affecting DC bias performance [9], when the compaction pressure is small, the particles do not undergo deformation and internal stress accumulation can limit the movement of magnetic domain walls and thus retard saturation magnetization and improve DC bias performance. When the pressure is increased above 1350 MPa, the density increases rapidly from 5.17 to 5.51 g cm^−3^, and the content of air gaps with high energy storage capacity decreases substantially. Moreover, the internal demagnetization field is weakened due to the deformation of particles, leading to the deterioration of the DC bias performance [16,33].

### 3.4. Effects of Annealing Temperatures on MPC Properties

**Figure 13:**
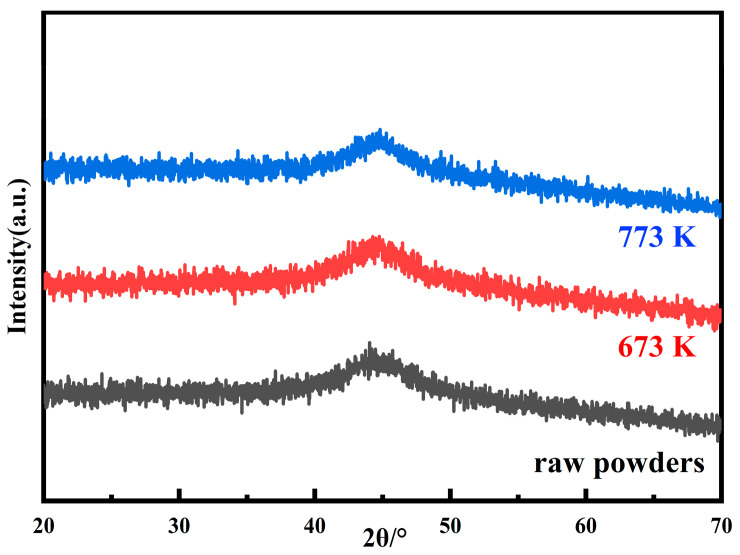
XRD patterns of raw powders and annealed powders at 673 and 773 K.

Heat treatment is an important step to improve the soft magnetic properties of the MPCs. The internal stress accumulated in the MPCs during the consolidation can be released by the annealing process, but attention should be paid to the reasonable selection of the annealing temperature [31]. An annealing temperature that is too high will lead to the crystallization of the amorphous matrix and the formation of coarse crystal phases, pinching the magnetic domain walls and deteriorating the soft magnetic properties. Simultaneously, organic insulating agents such as epoxy resins and phenolic resins will also decompose at high temperature, which significantly reduces the resistivity and greatly increases *P*_cv_. Therefore, the annealing temperature generally needs to be controlled below the crystallization temperature [18,30]. Combined with the DSC curve in Figure 4, five annealing temperatures of 423, 623, 673, 723, and 773 K were selected to study the effect of annealing temperatures on the performance of the MPCs. To verify whether crystallization occurs at the annealing temperatures set in this study, the raw powders were annealed at 673 and 773 K for 2 h, respectively, and the XRD patterns are shown in Figure 13. It can be seen that even at the highest annealing temperature of 773 K, the powders still remain completely amorphous, and it can be considered that the MPCs fabricated in this work are all AMPCs (amorphous magnetic powder cores).

**Figure 14:**
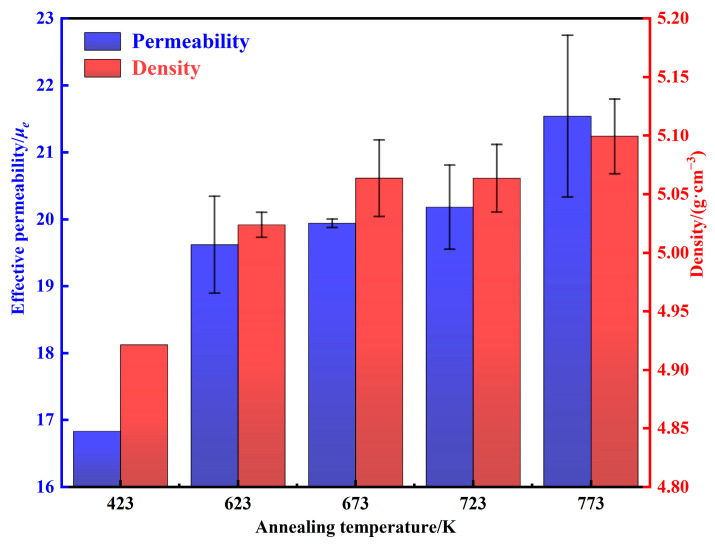
Density and permeability of AMPCs under different annealing temperatures.

The density and permeability of AMPCs annealing at different temperatures are shown in Figure 14. As the annealing temperature is increased from 423 to 773 K, the density of internal defects decreases, and the micro-scale cracks and pores gradually disappeared [20,32,34]. Therefore, the permeability and density are increased from 16.83 and 4.92 g·cm^−3^ to 21.54 ± 1.21 and 5.10 ± 0.03 g cm^−3^, respectively.

**Figure 15:**
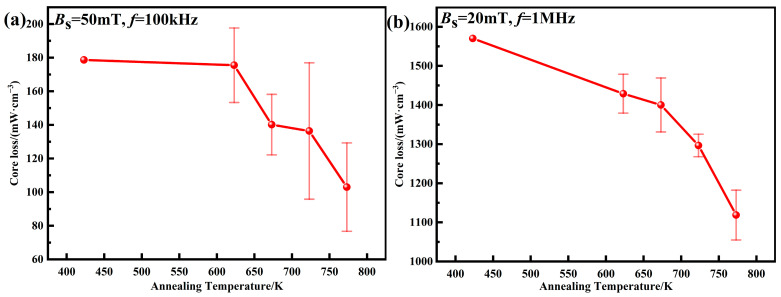
Core losses of AMPCs under different annealing temperatures: (**a**) *B*_s_ = 50 mT, *f* = 100 kHz; (**b**) *B*_s_ = 20 mT, *f* = 1 MHz.

The total core losses of AMPCs at different annealing temperatures are shown in Figure 15. When the annealing temperature is lower than 673 K, the internal stress is not fully released, and the decrease in *P*_cv_ is small. When the annealing temperature is increased from 673 to 773 K, the internal dislocation density of AMPCs decreases, the accumulated internal stress is fully released, and the uniformity of the phosphate insulating layers is improved [18,20]. Therefore, the core loss at *B*_m_ = 50 mT and *f* = 100 kHz is reduced from 140.2 ± 18.0 to 103.0 ± 26.3 mW·cm^–3^, and the core loss at *B*_m_ = 20 mT and *f* = 1 MHz is reduced from 1400.3 ± 69.1 to 1118.7 ± 63.7 mW cm^–3^.

**Figure 16:**
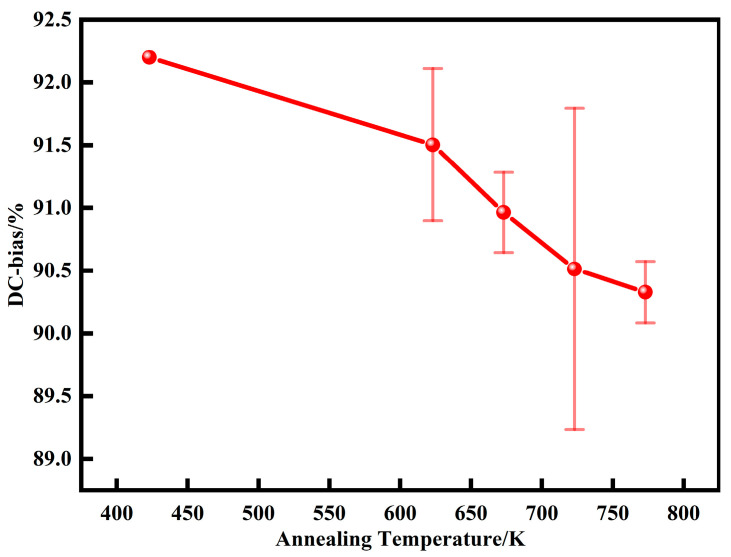
DC bias of AMPCs under different annealing temperatures (DC magnetic field is 71.25 Oe).

Figure 16 shows the DC bias performance of AMPCs at different annealing temperatures. With the increase of annealing temperatures, the DC-bias% gradually decreases. This is because during the annealing process, the internal stress is gradually released, the content of air gaps and the resistance to restrict the movement of the magnetic domain walls gradually decreases, and the material is more likely to reach saturation magnetization, and so the DC-bias% is reduced from 92.2 at 423 K to 90.3 ± 0.2 at 773 K.

Based on the optimization study of the above process parameters, the magnetic properties of MPCs fabricated in this study and previous studies are compared, as shown in Table 2. It can be seen that the AMPCs fabricated in this study have excellent DC bias performance and outstanding low core loss under high frequency, which are in line with the current development trends of high frequency, high efficiency, and integration of electronic components. The AMPCs can provide excellent raw material reserves for high-end inductor devices and are expected to be further used in 5G mobile communications, intelligent home electronics, and new energy automobiles.

## 4. Conclusions

In this study, Fe_70.8_Si_11.4_B_11.9_C_3.2_Cr_2.7_ (at. %) amorphous powders were produced by a novel gas–water combined atomization process, and corresponding magnetic powder cores were fabricated under different phosphating treatment, cold pressing, and heat treatment conditions. The main conclusions are as follows:

- The raw powders have a fine size of *d*_50_ = 28.87 μm and high circularity of 0.913, which is conducive to the forming of high-density MPCs and the enhancement of high-frequency properties; the powders exhibit excellent soft magnetic properties, which can provide an excellent raw material reserve for high-performance MPCs.
- With the increase of phosphating concentrations, the inhomogeneity of phosphate layers intensifies, and the content of non-ferromagnetic phases increases, and thus the permeability, density, and core losses deteriorate despite the enhancement of DC bias performance. With the increase of pressure, although the density increases, the excessive accumulation of internal stress makes the permeability increase and then decrease and the core losses increase continuously. When annealed below the crystallization temperature, the density of internal defects decreases as the annealing temperature increases, the permeability increases gradually, the core losses decrease, and the DC-bias% decreases due to the reduction in the content of air gaps.
- The AMPCs prepared by the optimized process of 0.4 wt.% phosphoric acid treatment, cold pressing at 550 MPa, and annealing at 773 K/2 h have excellent overall performance with the permeability of 21.54 ± 1.21, DC-bias% of 90.3 ± 0.2, and core losses of 103.0 ± 26.3 mW cm^−3^ and 1118.7 ± 63.7 mW cm^−3^ at 100 kHz/50 mT and 1 MHz/20 mT. The AMPCs fabricated in this work have outstanding DC bias and low core loss under high frequency.

## Figures and Tables

**Table 1:** Experimental schemes for FeSiBCCr AMPCs under different conditions.

Group	Phosphoric acid Concentration %	*P*_c_ MPa	*T*_a_ K
A	0.4	550	423
	0.6	550	423
	0.8	550	423
	1.2	550	423
	1.6	550	423
B	0.4	550	423
	0.4	950	423
	0.4	1150	423
	0.4	1300	423
	0.4	1900	423
	0.4	2350	423
C	0.4	550	423
	0.4	550	623
	0.4	550	673
	0.4	550	723
	0.4	550	773

**Table 2:** Comparison of magnetic properties between FeSiBCCr AMPCs in this work and the typical MPCs in previous studies.

Sample	*μ*_e_	Core Loss, *P*_cv_ (mW cm^−3^)		DC-Bias%	References
	100 kHz	100 kHz/0.05 T	1 MHz/0.02 T	71.25 Oe	
FeSiBCCr@phosphate@EP	21.5 ± 1.2	103.0 ± 26.3	1118.7 ± 63.7	90.3 ± 0.2	/
FeSiB + FeNi	30.1	314	/	/	[3]
FeSiCr@phosphate@polyimide	47.5	547	/	68.1	[10]
FeSiBPC@EP@Fe3O4	49.5	187	/	/	[15]
FeSiBPC@EP	44	301	/	/	[15]
FeSiCr@phosphate@EP	21.5	1016.8	/	89.4	[16]
FeSiBPNbCu@EP	63	475	/	81.2	[17]
FeSiCr@silicone resin	17.2	/	1442	/	[20]
FeSiCr + CIP@ silicone resin	19.7	/	1257	/	[20]
FeSiNi@phosphate@EP	/	643.2	/	74.4	[33]
FeSiPS + Al_2_O_3_@aluminum nitrate	36.49	398.5	/	/	[35]
FeSiBCCr@TiO_2_	67	265	/	75.4	[36]
FeSiBCCr@TiO_2_	74	301	/	80.0	[36]

## Data Availability

The data presented in this study are available on request from the corresponding author.
